# Poly[[μ-1,3-bis­(pyridin-3-yl)urea]bis­(μ_4_-succinato)dicopper(II)], a ribbon-like coordination polymer

**DOI:** 10.1107/S2414314623007472

**Published:** 2023-09-08

**Authors:** Frederick Ezenyilimba, Robert L. LaDuca

**Affiliations:** a E-35 Holmes Hall, Michigan State University, Lyman Briggs College, 919 E. Shaw Lane, East Lansing, MI 48825, USA; Benemérita Universidad Autónoma de Puebla, México

**Keywords:** crystal structure, coordination polymer, ribbon topology, copper, succinate

## Abstract

In the title com­pound, [Cu_2_(C_4_H_4_O_4_)_2_(C_11_H_10_N_4_O)]_*n*_, mono-periodic coordination polymer ribbons are held into the crystal structure by means of N—H⋯O hydrogen bonding and crystal packing forces.

## Abstract 2

A divalent copper one-dimensional ribbon copper(II) coordination polymer, with 1,3-bis­(pyridin-3-yl)urea and succinate ligands, was structurally characterized by single-crystal X-ray diffraction.

## Graphical abstract

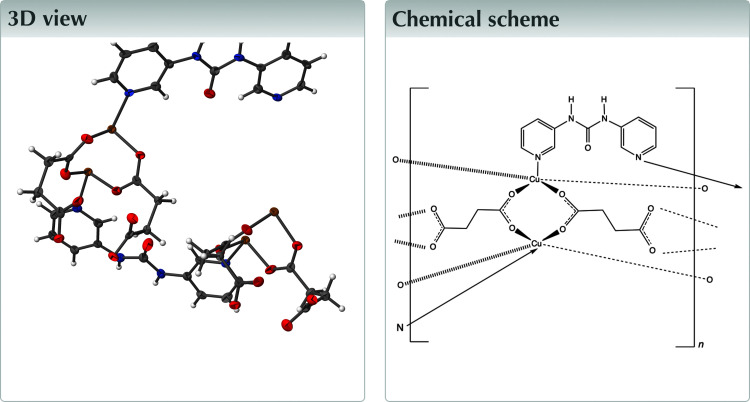

## Structure description

The title com­pound was isolated during an exploratory synthetic effort aiming to produce a copper coordination polymer containing both succinate (succ) and 1,3-bis­(pyridin-3-yl)urea (or 3,3′-di­pyridyl­urea, 3-dpu) ligands. Previously, our group had isolated a series of cadmium succinate coordination polymers featuring isomeric di­pyridyl­amide coligands. Structural topologies were highly dependent on the specific di­pyridyl­amide ligand used (Uebler *et al.*, 2013 ▸).

**Figure 1:**
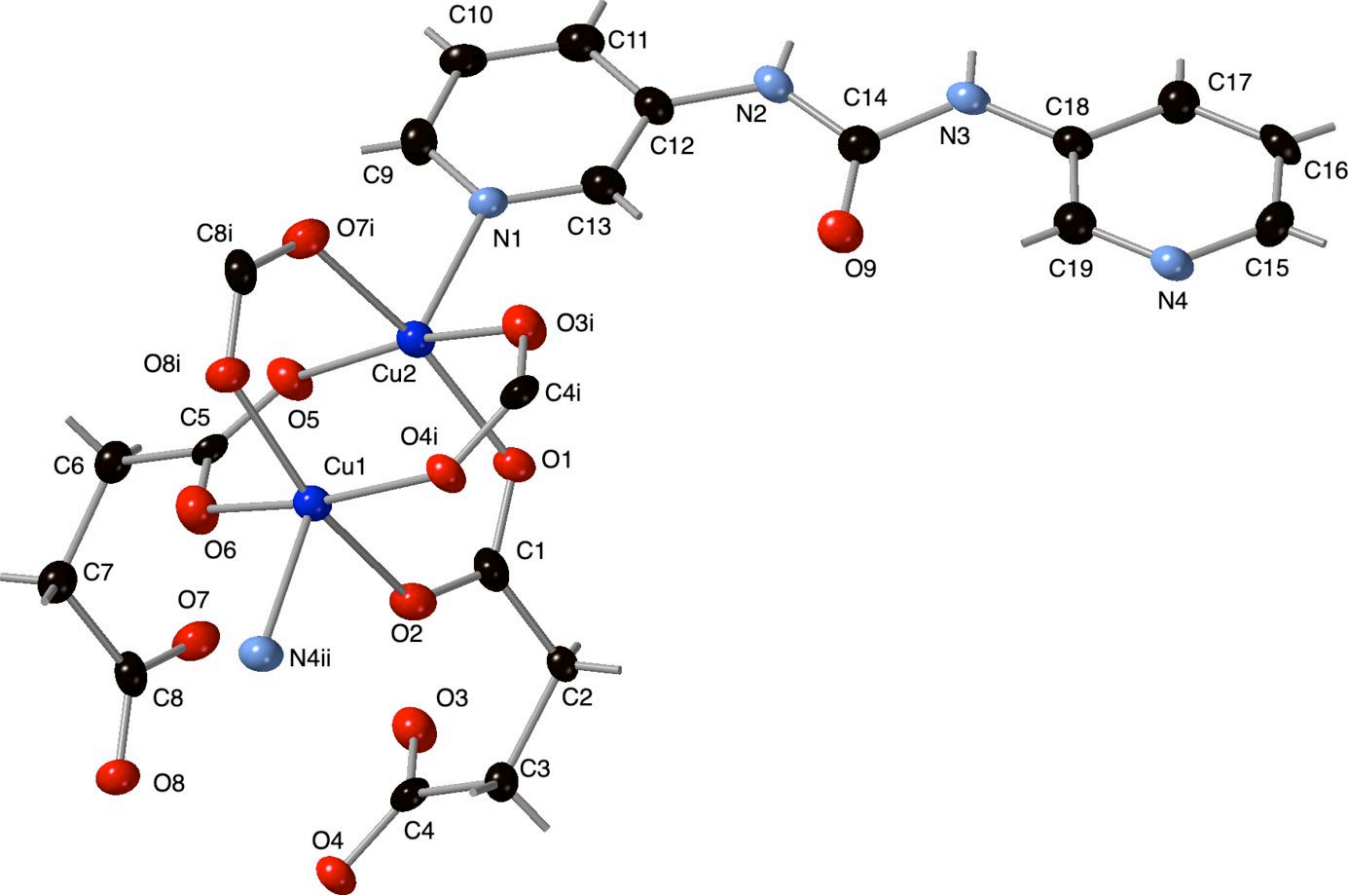
The copper coordination environments in the title com­pound with the full ligand set and the com­plete {Cu_2_(OCO)_4_} paddlewheel cluster. Displacement ellipsoids are drawn at the 50% probability level. Color code: Cu dark blue, O red, N light blue, and C black. H-atom positions are represented as sticks. The symmetry codes are as listed in Table 1 ▸.

The asymmetric unit of the title com­pound contains two divalent Cu atoms, two crystallographically distinct fully deprotonated succ ligands, and a full 3-dpu ligand. The Cu1 and Cu2 atoms display [NO_4_] square-pyramidal coordination environments, with elongated apical positions occupied by pyridyl N-atom donors from 3-dpu ligands. Their basal planes com­prise four carboxyl­ate O-atom donors from four different succ ligands (Table 1 ▸). The Cu1 and Cu2 atoms possess trigonality factors τ of 0.044 and 0.035 (Addison *et al.*, 1984 ▸), indicating only a slight variance from idealized square-pyramidal geometry. Complete coordination environments and ligand sets are shown in Fig. 1 ▸.

**Figure 2:**
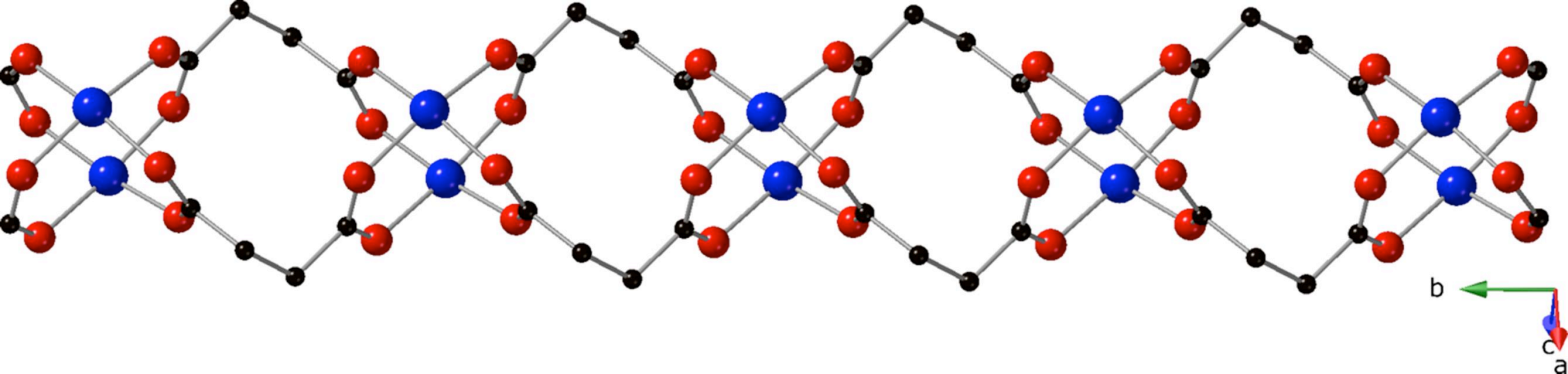
The [Cu_2_(succ)_2_]_*n*_ coordination polymer chain in the title com­pound, featuring {Cu_2_(OCO)_4_} paddlewheel clusters.

**Figure 3:**
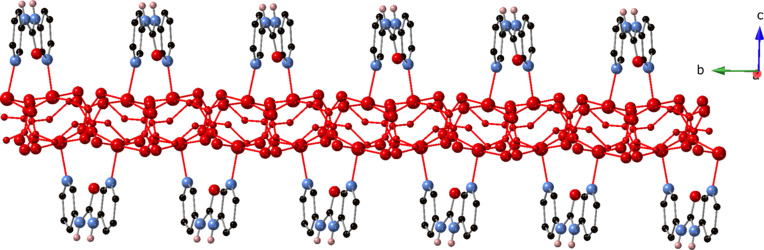
A [Cu_2_(succ)_2_(3-dpu)]_*n*_ coordination polymer ribbon in the title com­pound, with a [Cu_2_(succ)_2_]_*n*_ chain motif drawn in red.

The carboxyl­ate groups of the succ ligands bridge Cu1 and Cu2 atoms in a *syn*–*syn* fashion, giving rise to {Cu_2_(OCO)_4_} paddlewheel dimers with a Cu⋯Cu separation of 2.657 (1) Å. The full span of the *gauche*-conformation succ ligands connect the dimeric clusters into [Cu_2_(succ)_2_]_*n*_ coordination polymer chains oriented parallel to the *b* crystal direction (Fig. 2 ▸). The 3-dpu ligands, which adopt a *syn* conformation, conjoin Cu1 and Cu2 along the top and bottom of the [Cu_2_(succ)_2_]_*n*_ chain motifs, affording [Cu_2_(succ)_2_(3-dpu)]_*n*_ coordination polymer ribbons oriented parallel to the *b* crystal direction (Fig. 3 ▸).

**Figure 4:**
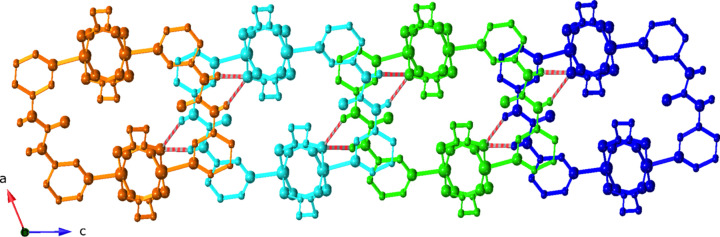
Supra­molecular layer formed by N—H⋯O hydrogen bonding (hatched bonds) between [Cu_2_(succ)_2_(3-dpu)]_*n*_ ribbon motifs.

**Figure 5:**
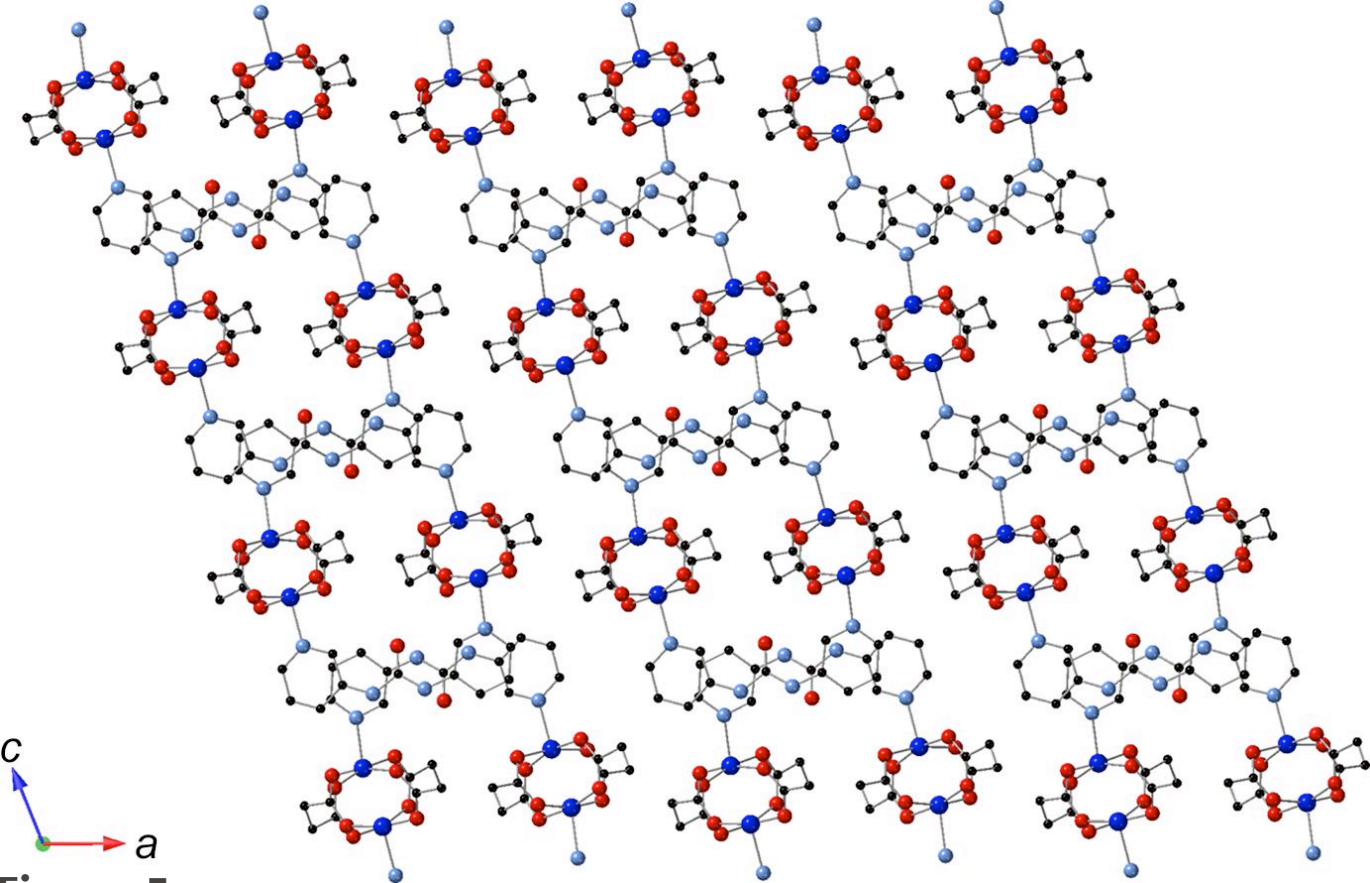
Aggregation of supra­molecular layer motifs in the title com­pound.

Regarding supra­molecular inter­actions, adjacent [Cu_2_(succ)_2_(3-dpu)]_*n*_ motifs aggregate into supra­molecular layers parallel to the *bc* crystal planes by means of N—H⋯O hydrogen bonding between the urea groups of 3-dpu ligands in one ribbon, and succ carboxyl­ate O atoms in the next ribbon (Fig. 4 ▸). In turn, the supra­molecular layers aggregate into the three-dimensional crystal structure of the title com­pound by crystal packing forces (Fig. 5 ▸). Details regarding the hydrogen bonding patterns in the title com­pound are listed in Table 2 ▸.

## Synthesis and crystallization

Cu(NO_3_)_2_·2.5H_2_O (86 mg, 0.37 mmol), succinic acid (succH_2_; 44 mg, 0.37 mmol), 3,3′-di­pyridyl­urea (3-dpu; 79 mg, 0.37 mmol), and 0.75 ml of a 1.0 *M* NaOH solution were placed into 10 ml distilled water in a Teflon-lined acid digestion bomb. The bomb was sealed and heated in an oven at 393 K for 48 h, and then cooled slowly to 273 K. Green crystals of the title com­plex were obtained in 43% yield.

## Refinement

Crystal data, data collection and structure refinement details are summarized in Table 3 ▸. All H atoms were placed in calculated positions and refined with a riding model.

## Supplementary Material

Crystal structure: contains datablock(s) I, global. DOI: 10.1107/S2414314623007472/bh4078sup1.cif

Structure factors: contains datablock(s) I. DOI: 10.1107/S2414314623007472/bh4078Isup2.hkl

CCDC reference: 2290886

Additional supporting information:  crystallographic information; 3D view; checkCIF report

## Figures and Tables

**Table 1:** Selected geometric parameters (Å, °)

Cu1—O2	1.960 (4)	Cu2—O1	1.979 (4)
Cu1—O4^i^	2.023 (4)	Cu2—O3^i^	1.990 (4)
Cu1—O6	1.963 (4)	Cu2—O5	1.980 (4)
Cu1—O8^i^	1.961 (4)	Cu2—O7^i^	1.973 (4)
Cu1—N4^ii^	2.197 (5)	Cu2—N1	2.167 (5)
			
O2—Cu1—O4^i^	90.48 (18)	O1—Cu2—O3^i^	89.16 (18)
O2—Cu1—O6	87.41 (18)	O1—Cu2—O5	90.34 (18)
O2—Cu1—O8^i^	166.45 (17)	O1—Cu2—N1	95.10 (17)
O2—Cu1—N4^ii^	91.11 (18)	O3^i^—Cu2—N1	99.11 (18)
O4^i^—Cu1—N4^ii^	89.25 (17)	O5—Cu2—O3^i^	166.52 (17)
O6—Cu1—O4^i^	169.11 (17)	O5—Cu2—N1	94.36 (18)
O6—Cu1—N4^ii^	101.47 (18)	O7^i^—Cu2—O1	168.62 (18)
O8^i^—Cu1—O4^i^	88.52 (17)	O7^i^—Cu2—O3^i^	88.50 (18)
O8^i^—Cu1—O6	91.03 (18)	O7^i^—Cu2—O5	89.35 (19)
O8^i^—Cu1—N4^ii^	102.38 (18)	O7^i^—Cu2—N1	96.27 (18)

Symmetry codes: (i) 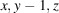 ; (ii) 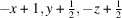 .

**Table 2:** Hydrogen-bond geometry (Å, °)

*D*—H⋯*A*	*D*—H	H⋯*A*	*D*⋯*A*	*D*—H⋯*A*
N2—H2⋯O4^iii^	0.88	2.21	3.042 (6)	157
N3—H3⋯O4^iii^	0.88	2.36	3.174 (6)	154

Symmetry code: (iii) 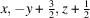 .

**Table 3:** Experimental details

Crystal data	
Chemical formula	[Cu_2_(C_4_H_4_O_4_)_2_(C_11_H_10_N_4_O)]
*M*_r_	573.45
Crystal system, space group	Monoclinic, *P*2_1_/*c*
Temperature (K)	173
*a*, *b*, *c* (Å)	15.587 (2), 6.7579 (10), 20.942 (3)
β (°)	111.614 (2)
*V* (Å^3^)	2050.9 (5)
*Z*	4
Radiation type	Mo *K*α
μ (mm^−1^)	2.14
Crystal size (mm)	0.24 × 0.12 × 0.05

Data collection	
Diffractometer	Bruker APEXII CCD
Absorption correction	Multi-scan (*SADABS*; Bruker, 2013 ▸)
*T*_min_, *T*_max_	0.568, 0.745
No. of measured, independent and observed [*I* > 2σ(*I*)] reflections	15941, 3758, 2378
*R*_int_	0.110
(sin θ/λ)_max_ (Å^−1^)	0.603

Refinement	
*R*[*F* ^2^ > 2σ(*F* ^2^)], *wR*(*F* ^2^), *S*	0.056, 0.151, 0.99
No. of reflections	3758
No. of parameters	307
H-atom treatment	H-atom parameters constrained
Δρ_max_, Δρ_min_ (e Å^−3^)	1.12, −0.60

Computer programs: *COSMO* (Bruker, 2009 ▸), *SAINT* (Bruker, 2013 ▸), *SHELXT2018* (Sheldrick, 2015*a* ▸), *SHELXL2018* (Sheldrick, 2015*b* ▸), *CrystalMakerX* (Palmer, 2020 ▸), and *OLEX2* (Dolomanov *et al.*, 2009 ▸).

## full crystallographic data

### Crystal data

**Table:**

[Cu_2_(C_4_H_4_O_4_)_2_(C_11_H_10_N_4_O)]	*F*(000) = 1160
*M**_r_* = 573.45	*D*_x_ = 1.857 Mg m^−^^3^
Monoclinic, *P*2_1_/*c*	Mo *K*α radiation, λ = 0.71073 Å
*a* = 15.587 (2) Å	Cell parameters from 2446 reflections
*b* = 6.7579 (10) Å	θ = 2.8–25.1°
*c* = 20.942 (3) Å	µ = 2.14 mm^−^^1^
β = 111.614 (2)°	*T* = 173 K
*V* = 2050.9 (5) Å^3^	Plate, green
*Z* = 4	0.24 × 0.12 × 0.05 mm

### Data collection

**Table:**

Bruker APEXII CCD diffractometer	3758 independent reflections
Radiation source: sealed tube	2378 reflections with *I* > 2σ(*I*)
Graphite monochromator	*R*_int_ = 0.110
Detector resolution: 8.36 pixels mm^-1^	θ_max_ = 25.4°, θ_min_ = 1.4°
ω scans	*h* = −18→18
Absorption correction: multi-scan (SADABS; Bruker, 2013)	*k* = −8→8
*T*_min_ = 0.568, *T*_max_ = 0.745	*l* = −25→25
15941 measured reflections	

### Refinement

**Table:**

Refinement on *F*^2^	Primary atom site location: dual
Least-squares matrix: full	Secondary atom site location: difference Fourier map
*R*[*F*^2^ > 2σ(*F*^2^)] = 0.056	Hydrogen site location: inferred from neighbouring sites
*wR*(*F*^2^) = 0.151	H-atom parameters constrained
*S* = 0.99	*w* = 1/[σ^2^(*F*_o_^2^) + (0.0685*P*)^2^] where *P* = (*F*_o_^2^ + 2*F*_c_^2^)/3
3758 reflections	(Δ/σ)_max_ = 0.001
307 parameters	Δρ_max_ = 1.12 e Å^−^^3^
0 restraints	Δρ_min_ = −0.60 e Å^−^^3^

### Special details

**Table:**

Experimental. Data was collected using a BRUKER CCD (charge coupled device) based diffractometer equipped with an Oxford low-temperature apparatus operating at 173 K. A suitable crystal was chosen and mounted on a nylon loop using Paratone oil. Data were measured using omega scans of 0.5° per frame for 30 s. The total number of images were based on results from the program COSMO where redundancy was expected to be 4 and completeness to 0.83Å to 100%. Cell parameters were retrieved using APEX II software and refined using SAINT on all observed reflections.Data reduction was performed using the SAINT software which corrects for Lp. Scaling and absorption corrections were applied using SADABS6 multi-scan technique, supplied by George Sheldrick. The structure was solved by the direct method using the SHELXT program and refined by least squares method on F2, SHELXL, incorporated in OLEX2.

### Fractional atomic coordinates and isotropic or equivalent isotropic displacement parameters (Å^2^)

**Table:**

	*x*	*y*	*z*	*U*_iso_*/*U*_eq_	
Cu1	0.76095 (5)	0.56430 (10)	0.20555 (4)	0.0199 (2)	
Cu2	0.77124 (5)	0.49295 (10)	0.33295 (4)	0.0198 (2)	
O1	0.6695 (3)	0.6855 (6)	0.3154 (2)	0.0241 (10)	
O2	0.6730 (3)	0.7626 (6)	0.2124 (2)	0.0257 (11)	
O3	0.6784 (3)	1.2834 (6)	0.2892 (2)	0.0250 (10)	
O4	0.6616 (3)	1.3542 (6)	0.1809 (2)	0.0228 (10)	
O5	0.8617 (3)	0.7122 (6)	0.3540 (2)	0.0272 (11)	
O6	0.8587 (3)	0.7601 (6)	0.2474 (2)	0.0269 (11)	
O7	0.8683 (3)	1.3085 (6)	0.3309 (2)	0.0284 (11)	
O8	0.8527 (3)	1.3529 (6)	0.2213 (2)	0.0232 (10)	
O9	0.5334 (3)	0.3287 (7)	0.4400 (2)	0.0355 (12)	
N1	0.7897 (3)	0.4419 (7)	0.4394 (3)	0.0190 (12)	
N2	0.6521 (4)	0.3177 (7)	0.5442 (3)	0.0226 (12)	
H2	0.665061	0.298117	0.588321	0.027*	
N3	0.5065 (4)	0.2208 (7)	0.5341 (3)	0.0225 (12)	
H3	0.533315	0.195629	0.578293	0.027*	
N4	0.2765 (4)	0.1280 (7)	0.4043 (2)	0.0207 (12)	
C1	0.6429 (4)	0.7848 (8)	0.2603 (3)	0.0196 (14)	
C2	0.5697 (4)	0.9394 (8)	0.2500 (3)	0.0194 (14)	
H2A	0.579259	1.002925	0.294759	0.023*	
H2B	0.508546	0.874023	0.234080	0.023*	
C3	0.5692 (4)	1.0992 (9)	0.1984 (3)	0.0190 (14)	
H3A	0.576689	1.034695	0.158311	0.023*	
H3B	0.508195	1.165147	0.181940	0.023*	
C4	0.6433 (4)	1.2555 (8)	0.2259 (3)	0.0181 (14)	
C5	0.8876 (4)	0.7960 (9)	0.3111 (3)	0.0197 (14)	
C6	0.9644 (4)	0.9500 (8)	0.3372 (3)	0.0225 (15)	
H6A	0.961873	1.010462	0.379568	0.027*	
H6B	1.024551	0.881653	0.349782	0.027*	
C7	0.9604 (4)	1.1147 (9)	0.2866 (3)	0.0240 (15)	
H7A	1.021401	1.180573	0.301650	0.029*	
H7B	0.948973	1.054800	0.241052	0.029*	
C8	0.8870 (4)	1.2704 (9)	0.2789 (3)	0.0213 (15)	
C9	0.8712 (5)	0.4774 (9)	0.4890 (3)	0.0280 (16)	
H9	0.922054	0.513711	0.476843	0.034*	
C10	0.8834 (5)	0.4625 (9)	0.5578 (3)	0.0279 (16)	
H10	0.942091	0.487920	0.592295	0.034*	
C11	0.8108 (4)	0.4111 (9)	0.5757 (3)	0.0250 (15)	
H11	0.818417	0.401257	0.622687	0.030*	
C12	0.7253 (4)	0.3733 (8)	0.5245 (3)	0.0213 (14)	
C13	0.7181 (5)	0.3899 (9)	0.4566 (3)	0.0234 (15)	
H13	0.660291	0.363379	0.421060	0.028*	
C14	0.5621 (5)	0.2911 (9)	0.5012 (3)	0.0235 (15)	
C15	0.2276 (5)	0.0979 (9)	0.4441 (4)	0.0260 (16)	
H15	0.163956	0.066116	0.423285	0.031*	
C16	0.2677 (5)	0.1121 (8)	0.5150 (3)	0.0253 (15)	
H16	0.231447	0.093317	0.542417	0.030*	
C17	0.3608 (4)	0.1539 (9)	0.5456 (3)	0.0249 (15)	
H17	0.389672	0.160856	0.594176	0.030*	
C18	0.4114 (4)	0.1852 (8)	0.5044 (3)	0.0188 (14)	
C19	0.3656 (5)	0.1700 (9)	0.4340 (3)	0.0251 (15)	
H19	0.399984	0.190872	0.405368	0.030*	

### Atomic displacement parameters (Å^2^)

**Table:**

	*U*^11^	*U*^22^	*U*^33^	*U*^12^	*U*^13^	*U*^23^
Cu1	0.0226 (5)	0.0173 (4)	0.0204 (4)	0.0010 (3)	0.0088 (4)	0.0007 (3)
Cu2	0.0227 (5)	0.0170 (4)	0.0207 (4)	0.0002 (3)	0.0092 (4)	0.0002 (3)
O1	0.030 (3)	0.021 (2)	0.025 (3)	0.0066 (19)	0.015 (2)	0.0068 (19)
O2	0.034 (3)	0.021 (2)	0.025 (3)	0.009 (2)	0.015 (2)	0.0029 (19)
O3	0.031 (3)	0.023 (2)	0.022 (3)	−0.007 (2)	0.010 (2)	−0.0049 (19)
O4	0.026 (3)	0.024 (2)	0.018 (2)	−0.0066 (19)	0.008 (2)	0.0026 (19)
O5	0.032 (3)	0.022 (2)	0.025 (3)	−0.012 (2)	0.007 (2)	0.001 (2)
O6	0.032 (3)	0.024 (3)	0.028 (3)	−0.006 (2)	0.015 (2)	−0.006 (2)
O7	0.029 (3)	0.026 (3)	0.032 (3)	0.012 (2)	0.012 (2)	0.003 (2)
O8	0.025 (3)	0.022 (2)	0.021 (2)	0.0053 (19)	0.007 (2)	0.0023 (19)
O9	0.028 (3)	0.049 (3)	0.027 (3)	−0.003 (2)	0.006 (2)	0.013 (2)
N1	0.019 (3)	0.015 (3)	0.019 (3)	0.002 (2)	0.004 (2)	0.002 (2)
N2	0.029 (3)	0.019 (3)	0.024 (3)	0.001 (2)	0.015 (3)	0.004 (2)
N3	0.029 (3)	0.017 (3)	0.021 (3)	0.001 (2)	0.009 (3)	0.005 (2)
N4	0.028 (3)	0.015 (3)	0.019 (3)	−0.002 (2)	0.008 (3)	0.001 (2)
C1	0.024 (4)	0.012 (3)	0.025 (4)	−0.009 (3)	0.012 (3)	−0.006 (3)
C2	0.018 (3)	0.018 (3)	0.024 (3)	−0.002 (3)	0.010 (3)	0.004 (3)
C3	0.018 (3)	0.020 (3)	0.021 (3)	−0.001 (3)	0.009 (3)	−0.002 (3)
C4	0.016 (3)	0.010 (3)	0.026 (4)	0.005 (2)	0.005 (3)	−0.002 (3)
C5	0.013 (3)	0.020 (3)	0.024 (4)	0.005 (3)	0.003 (3)	0.005 (3)
C6	0.017 (4)	0.020 (4)	0.028 (4)	−0.004 (3)	0.005 (3)	−0.003 (3)
C7	0.021 (4)	0.023 (4)	0.028 (4)	0.001 (3)	0.009 (3)	−0.002 (3)
C8	0.021 (4)	0.016 (3)	0.032 (4)	−0.007 (3)	0.014 (3)	−0.004 (3)
C9	0.023 (4)	0.036 (4)	0.028 (4)	−0.001 (3)	0.013 (3)	0.001 (3)
C10	0.024 (4)	0.029 (4)	0.021 (4)	−0.001 (3)	−0.002 (3)	0.008 (3)
C11	0.032 (4)	0.026 (4)	0.017 (3)	0.006 (3)	0.009 (3)	0.000 (3)
C12	0.028 (4)	0.012 (3)	0.027 (4)	−0.001 (3)	0.014 (3)	0.001 (3)
C13	0.031 (4)	0.021 (4)	0.019 (3)	0.002 (3)	0.011 (3)	−0.001 (3)
C14	0.027 (4)	0.017 (3)	0.029 (4)	0.003 (3)	0.012 (3)	0.002 (3)
C15	0.019 (4)	0.019 (4)	0.040 (4)	0.002 (3)	0.011 (3)	−0.003 (3)
C16	0.038 (4)	0.014 (3)	0.033 (4)	−0.001 (3)	0.024 (4)	0.005 (3)
C17	0.026 (4)	0.026 (4)	0.024 (4)	0.001 (3)	0.011 (3)	−0.002 (3)
C18	0.024 (4)	0.015 (3)	0.018 (3)	0.000 (3)	0.008 (3)	0.001 (3)
C19	0.029 (4)	0.021 (4)	0.027 (4)	0.001 (3)	0.013 (3)	−0.001 (3)

### Geometric parameters (Å, º)

**Table:**

Cu1—O2	1.960 (4)	C2—H2A	0.9900
Cu1—O4^i^	2.023 (4)	C2—H2B	0.9900
Cu1—O6	1.963 (4)	C2—C3	1.526 (8)
Cu1—O8^i^	1.961 (4)	C3—H3A	0.9900
Cu1—N4^ii^	2.197 (5)	C3—H3B	0.9900
Cu2—O1	1.979 (4)	C3—C4	1.515 (8)
Cu2—O3^i^	1.990 (4)	C5—C6	1.528 (8)
Cu2—O5	1.980 (4)	C6—H6A	0.9900
Cu2—O7^i^	1.973 (4)	C6—H6B	0.9900
Cu2—N1	2.167 (5)	C6—C7	1.522 (8)
O1—C1	1.265 (7)	C7—H7A	0.9900
O2—C1	1.264 (7)	C7—H7B	0.9900
O3—C4	1.248 (7)	C7—C8	1.519 (8)
O4—C4	1.271 (7)	C9—H9	0.9500
O5—C5	1.249 (7)	C9—C10	1.386 (9)
O6—C5	1.263 (7)	C10—H10	0.9500
O7—C8	1.254 (7)	C10—C11	1.361 (9)
O8—C8	1.256 (7)	C11—H11	0.9500
O9—C14	1.218 (7)	C11—C12	1.392 (8)
N1—C9	1.333 (8)	C12—C13	1.389 (8)
N1—C13	1.340 (7)	C13—H13	0.9500
N2—H2	0.8800	C15—H15	0.9500
N2—C12	1.401 (8)	C15—C16	1.387 (9)
N2—C14	1.371 (8)	C16—H16	0.9500
N3—H3	0.8800	C16—C17	1.383 (9)
N3—C14	1.376 (8)	C17—H17	0.9500
N3—C18	1.400 (8)	C17—C18	1.383 (8)
N4—C15	1.335 (8)	C18—C19	1.385 (8)
N4—C19	1.327 (8)	C19—H19	0.9500
C1—C2	1.504 (8)		
			
O2—Cu1—O4^i^	90.48 (18)	O3—C4—O4	125.4 (5)
O2—Cu1—O6	87.41 (18)	O3—C4—C3	119.0 (5)
O2—Cu1—O8^i^	166.45 (17)	O4—C4—C3	115.5 (5)
O2—Cu1—N4^ii^	91.11 (18)	O5—C5—O6	126.1 (6)
O4^i^—Cu1—N4^ii^	89.25 (17)	O5—C5—C6	117.9 (6)
O6—Cu1—O4^i^	169.11 (17)	O6—C5—C6	115.9 (5)
O6—Cu1—N4^ii^	101.47 (18)	C5—C6—H6A	108.5
O8^i^—Cu1—O4^i^	88.52 (17)	C5—C6—H6B	108.5
O8^i^—Cu1—O6	91.03 (18)	H6A—C6—H6B	107.5
O8^i^—Cu1—N4^ii^	102.38 (18)	C7—C6—C5	115.0 (5)
O1—Cu2—O3^i^	89.16 (18)	C7—C6—H6A	108.5
O1—Cu2—O5	90.34 (18)	C7—C6—H6B	108.5
O1—Cu2—N1	95.10 (17)	C6—C7—H7A	108.6
O3^i^—Cu2—N1	99.11 (18)	C6—C7—H7B	108.6
O5—Cu2—O3^i^	166.52 (17)	H7A—C7—H7B	107.6
O5—Cu2—N1	94.36 (18)	C8—C7—C6	114.6 (5)
O7^i^—Cu2—O1	168.62 (18)	C8—C7—H7A	108.6
O7^i^—Cu2—O3^i^	88.50 (18)	C8—C7—H7B	108.6
O7^i^—Cu2—O5	89.35 (19)	O7—C8—O8	126.2 (6)
O7^i^—Cu2—N1	96.27 (18)	O7—C8—C7	117.2 (6)
C1—O1—Cu2	119.2 (4)	O8—C8—C7	116.6 (5)
C1—O2—Cu1	127.8 (4)	N1—C9—H9	119.2
C4—O3—Cu2^iii^	123.8 (4)	N1—C9—C10	121.6 (6)
C4—O4—Cu1^iii^	122.6 (4)	C10—C9—H9	119.2
C5—O5—Cu2	124.8 (4)	C9—C10—H10	120.2
C5—O6—Cu1	121.1 (4)	C11—C10—C9	119.7 (6)
C8—O7—Cu2^iii^	125.0 (4)	C11—C10—H10	120.2
C8—O8—Cu1^iii^	120.7 (4)	C10—C11—H11	120.3
C9—N1—Cu2	120.0 (4)	C10—C11—C12	119.5 (6)
C9—N1—C13	119.1 (5)	C12—C11—H11	120.3
C13—N1—Cu2	120.7 (4)	C11—C12—N2	118.4 (6)
C12—N2—H2	116.9	C13—C12—N2	123.7 (6)
C14—N2—H2	116.9	C13—C12—C11	117.9 (6)
C14—N2—C12	126.1 (5)	N1—C13—C12	122.3 (6)
C14—N3—H3	116.7	N1—C13—H13	118.8
C14—N3—C18	126.5 (5)	C12—C13—H13	118.8
C18—N3—H3	116.7	O9—C14—N2	123.6 (6)
C15—N4—Cu1^iv^	129.1 (4)	O9—C14—N3	122.9 (6)
C19—N4—Cu1^iv^	111.2 (4)	N2—C14—N3	113.4 (6)
C19—N4—C15	118.6 (6)	N4—C15—H15	119.2
O1—C1—C2	118.4 (5)	N4—C15—C16	121.5 (6)
O2—C1—O1	124.7 (6)	C16—C15—H15	119.2
O2—C1—C2	116.9 (5)	C15—C16—H16	120.3
C1—C2—H2A	108.9	C17—C16—C15	119.4 (6)
C1—C2—H2B	108.9	C17—C16—H16	120.3
C1—C2—C3	113.5 (5)	C16—C17—H17	120.5
H2A—C2—H2B	107.7	C18—C17—C16	119.0 (6)
C3—C2—H2A	108.9	C18—C17—H17	120.5
C3—C2—H2B	108.9	C17—C18—N3	120.1 (6)
C2—C3—H3A	108.6	C17—C18—C19	117.7 (6)
C2—C3—H3B	108.6	C19—C18—N3	122.1 (6)
H3A—C3—H3B	107.5	N4—C19—C18	123.7 (6)
C4—C3—C2	114.8 (5)	N4—C19—H19	118.2
C4—C3—H3A	108.6	C18—C19—H19	118.2
C4—C3—H3B	108.6		
			
Cu1—O2—C1—O1	4.2 (9)	N4—C15—C16—C17	−1.5 (9)
Cu1—O2—C1—C2	−175.2 (4)	C1—C2—C3—C4	−78.3 (7)
Cu1^iii^—O4—C4—O3	5.2 (8)	C2—C3—C4—O3	−22.2 (8)
Cu1^iii^—O4—C4—C3	−177.9 (4)	C2—C3—C4—O4	160.7 (5)
Cu1—O6—C5—O5	2.4 (9)	C5—C6—C7—C8	75.8 (7)
Cu1—O6—C5—C6	179.4 (4)	C6—C7—C8—O7	32.4 (8)
Cu1^iii^—O8—C8—O7	0.4 (9)	C6—C7—C8—O8	−149.6 (5)
Cu1^iii^—O8—C8—C7	−177.5 (4)	C9—N1—C13—C12	0.7 (9)
Cu1^iv^—N4—C15—C16	167.8 (4)	C9—C10—C11—C12	0.3 (9)
Cu1^iv^—N4—C19—C18	−169.4 (5)	C10—C11—C12—N2	179.0 (6)
Cu2—O1—C1—O2	4.5 (8)	C10—C11—C12—C13	0.0 (9)
Cu2—O1—C1—C2	−176.2 (4)	C11—C12—C13—N1	−0.5 (9)
Cu2^iii^—O3—C4—O4	0.4 (8)	C12—N2—C14—O9	−6.2 (9)
Cu2^iii^—O3—C4—C3	−176.4 (4)	C12—N2—C14—N3	175.0 (5)
Cu2—O5—C5—O6	2.4 (9)	C13—N1—C9—C10	−0.3 (9)
Cu2—O5—C5—C6	−174.6 (4)	C14—N2—C12—C11	174.2 (5)
Cu2^iii^—O7—C8—O8	5.8 (9)	C14—N2—C12—C13	−6.8 (9)
Cu2^iii^—O7—C8—C7	−176.4 (4)	C14—N3—C18—C17	−166.6 (6)
Cu2—N1—C9—C10	174.5 (5)	C14—N3—C18—C19	16.7 (9)
Cu2—N1—C13—C12	−174.1 (4)	C15—N4—C19—C18	−0.2 (9)
O1—C1—C2—C3	157.7 (5)	C15—C16—C17—C18	1.6 (9)
O2—C1—C2—C3	−22.9 (8)	C16—C17—C18—N3	−177.7 (5)
O5—C5—C6—C7	−150.1 (6)	C16—C17—C18—C19	−0.9 (9)
O6—C5—C6—C7	32.6 (8)	C17—C18—C19—N4	0.3 (9)
N1—C9—C10—C11	−0.2 (10)	C18—N3—C14—O9	−1.0 (10)
N2—C12—C13—N1	−179.5 (5)	C18—N3—C14—N2	177.8 (5)
N3—C18—C19—N4	177.0 (5)	C19—N4—C15—C16	0.8 (9)

Symmetry codes: (i) *x*, *y*−1, *z*; (ii) −*x*+1, *y*+1/2, −*z*+1/2; (iii) *x*, *y*+1, *z*; (iv) −*x*+1, *y*−1/2, −*z*+1/2.

### Hydrogen-bond geometry (Å, º)

**Table:**

*D*—H···*A*	*D*—H	H···*A*	*D*···*A*	*D*—H···*A*
N2—H2···O4^v^	0.88	2.21	3.042 (6)	157
N3—H3···O4^v^	0.88	2.36	3.174 (6)	154
C2—H2*A*···O3	0.99	2.48	2.815 (7)	100
C3—H3*A*···O2	0.99	2.38	2.744 (7)	101
C6—H6*A*···O7	0.99	2.47	2.825 (7)	100
C7—H7*B*···O6	0.99	2.47	2.824 (8)	101
C9—H9···O5	0.95	2.74	3.196 (8)	110
C13—H13···O9	0.95	2.17	2.800 (8)	123
C19—H19···O2^iv^	0.95	2.35	2.965 (8)	122
C19—H19···O4^vi^	0.95	2.84	3.126 (7)	98
C19—H19···O9	0.95	2.15	2.786 (8)	124

Symmetry codes: (iv) −*x*+1, *y*−1/2, −*z*+1/2; (v) *x*, −*y*+3/2, *z*+1/2; (vi) −*x*+1, *y*−3/2, −*z*+1/2.
